# Malaria and large dams in sub-Saharan Africa: future impacts in a changing climate

**DOI:** 10.1186/s12936-016-1498-9

**Published:** 2016-09-05

**Authors:** Solomon Kibret, Jonathan Lautze, Matthew McCartney, Luxon Nhamo, G. Glenn Wilson

**Affiliations:** 1 Program in Public Health, University of California Irvine, Irvine, CA 92697 USA; 2 International Water Management Institute, Pretoria, South Africa; 3 International Water Management Institute, Vientiane, Lao People’s Democratic Republic; 4 Ecosystem Management, School of Environmental and Rural Science, University of New England, Armidale, NSW 2351 Australia

**Keywords:** Malaria, Dam, Reservoir, Climate scenario, Sub-Saharan Africa

## Abstract

**Background:**

Sub-Saharan Africa (SSA) has embarked on a new era of dam building to improve food security and promote economic development. Nonetheless, the future impacts of dams on malaria transmission are poorly understood and seldom investigated in the context of climate and demographic change.

**Methods:**

The distribution of malaria in the vicinity of 1268 existing dams in SSA was mapped under the Intergovernmental Panel on Climate Change (IPCC) representative concentration pathways (RCP) 2.6 and 8.5. Population projections and malaria incidence estimates were used to compute population at risk of malaria in both RCPs. Assuming no change in socio-economic interventions that may mitigate impacts, the change in malaria stability and malaria burden in the vicinity of the dams was calculated for the two RCPs through to the 2080s. Results were compared against the 2010 baseline. The annual number of malaria cases associated with dams and climate change was determined for each of the RCPs.

**Results:**

The number of dams located in malarious areas is projected to increase in both RCPs. Population growth will add to the risk of transmission. The population at risk of malaria around existing dams and associated reservoirs, is estimated to increase from 15 million in 2010 to 21–23 million in the 2020s, 25–26 million in the 2050s and 28–29 million in the 2080s, depending on RCP. The number of malaria cases associated with dams in malarious areas is expected to increase from 1.1 million in 2010 to 1.2–1.6 million in the 2020s, 2.1–3.0 million in the 2050s and 2.4–3.0 million in the 2080s depending on RCP. The number of cases will always be higher in RCP 8.5 than RCP 2.6.

**Conclusion:**

In the absence of changes in other factors that affect transmission (e.g., socio-economic), the impact of dams on malaria in SSA will be significantly exacerbated by climate change and increases in population. Areas without malaria transmission at present, which will transition to regions of unstable transmission, may be worst affected. Modifying conventional water management frameworks to improve malaria control, holds the potential to mitigate some of this increase and should be more actively implemented.

**Electronic supplementary material:**

The online version of this article (doi:10.1186/s12936-016-1498-9) contains supplementary material, which is available to authorized users.

## Background

### Dams are widely associated with malaria

More than 2000 large dams have been built and over 200 are currently under construction or planned in sub-Saharan Africa (SSA), with the aim of enhancing food security, increasing hydropower generation, managing variability and promoting economic growth [1]. Despite these social and economic benefits, there is a growing body of evidence that dams frequently intensify malaria transmission in SSA. For instance, increased malaria has been documented following the construction of the Bamendjin Dam in Cameroon [2], the Kamburu Dam in Kenya [3], the Koka reservoir in central Ethiopia [4, 5], the Gilgel Gibe Dam in southwest Ethiopia [6], the Manyuchi Dam in Zimbabwe [7], the Diama Dam in Senegal [8], and the Akosombo Dam in Ghana [9]. The overall malaria impact of dams at regional scale has also been assessed. Keiser et al. [10] postulated that 3.1 million people were at risk of malaria due to dams in SSA. Using a more extensive data set, Kibret et al. [11] estimated that more than 15 million people were at risk and that 1.1 million cases a year were associated with 1268 large dams in SSA.

### Dam-malaria impacts do not occur in a vacuum

The distribution and intensity of malaria is influenced by ecological, entomological and climatic factors through their influence on both the vector and the parasite [12]. Rainfall is the ultimate control on the availability of breeding habitat for mosquito vectors [13], and temperature determines the length of development of larval mosquitoes and malaria parasites [14], as well as the lifespan and the rate of blood feeding of adult vectors [15, 16]. Dams must, therefore, be considered in the context of these factors to fully understand their impact. A recent review, for example, indicated that the degree to which dams enhance malaria in SSA is in part a function of malaria transmission stability. It found that dams significantly increased malaria in areas of unstable (seasonal) transmission but had a much lower effect in areas of stable (perennial) transmission [17].

### Climate change may alter dams’ impact on malaria

Climate change will increase temperature and modify rainfall patterns in SSA [18]. Although early studies predicted widespread increase in malaria transmission due to climate change [19–23], more recent literature suggest a shift in distribution rather than a large net increase [24–26]. For instance, a recent report indicated that future climate might render the tropical highland regions more suitable for malaria transmission [27]. However, the likely consequences of climate change remain uncertain, in part because disease transmission is also determined by non-climate factors (e.g., malaria control activities, access to health care, socio-economic, environmental and behavioural factors) that can exacerbate or negate climatic influences [28–30]. Furthermore, there exists an ongoing debate on the level of uncertainty of future climate projections and their biophysical impacts, which result from uncertainty in both the greenhouse gas emission trajectory and the results of model simulations [18].

### No study has examined future impacts of dams in light of climate change

While there is one important body of work that examines dams and malaria in Africa [11] and a growing body of work assessing climate change and malaria [19–30], there has been no study to date that explicitly examines future malaria transmission around dams in light of climate change. As water storage provides potential mosquito breeding habitats and climate determines the development of mosquitoes and malaria parasites, it is important to understand how their interaction affects malaria transmission in the context of changing climate. This knowledge will help to devise optimal interventions and allocation of resources to fight disease transmission around dams.

### Estimating impact

The objective of the present study was to assess future malaria transmission in the vicinity of reservoirs in light of climatic change and projected population increases. For RCP 2.6 and RCP 8.5, the number of dams, population at risk and malaria cases in stable and unstable areas were estimated for future time intervals: the 2020s (i.e., 2020–2029), 2050s (i.e., 2050–2059) and 2080s (i.e., 2080–2089). Finally, the number of malaria cases associated with dams, climate change and population growth were determined separately for each time interval. This study is an extension of the previously published study [11].

## Methods

### Study area

The study was conducted in SSA. This region accounts for 90 % of the global malaria burden, i.e., 174 million cases annually [31]. Currently, malaria transmission is generally stable in western and central Africa, unstable in much of eastern Africa and unstable or absent in southern Africa [32]. In SSA, intensive malaria control efforts since the beginning of the twenty-first century, have resulted in a reduction of malaria-associated mortality by 45 % and 1.6 million fewer malaria deaths occurred between 2001 and 2015 [33]. Encouraged by this progress, the World Health Organization (WHO) has set ambitious goals to reduce the global malaria burden by 90 % by 2030 when compared to 2015 [33].

*Plasmodium falciparum* is the most prevalent and most fatal malaria parasite in the region [31]. *Anopheles gambiae* and *Anopheles funestus* are the predominant malaria vector species in wet and humid areas, while *Anopheles arabiensis* is the most common vector in drier climates [34, 35]. The variation in intensity of malaria transmission across SSA is partly the result of the relative abundance of these species across different ecological settings [36].

### Data source

The present study used four major data sources: (1) previously published global maps that show the future distribution of malaria under RCP 2.6 and 8.5 [27]; (2) databases of currently existing large dams; (3) projected population for the 2020s, 2050s and 2080s; and, (4) the Malaria Atlas Project (MAP) database of malaria incidence. These were used to estimate the population at risk around dams in areas of stable and unstable malaria transmission and to determine the possible impact of climate change on malaria transmission in the vicinity of reservoirs behind large dams.

### Data on existing African dams

The distribution of existing dams in SSA was mapped using the geo-referenced locations of individual dams in the Food and Agriculture Organization (FAO) African dam database [37] and the International Rivers database [38]. Data on water storage capacity, dam height and reservoir surface area were obtained from the World Register of Dams [39] and the Global Reservoirs and Dams (GRanD) database [40]. Locations and parameters of additional dams were obtained from a number of journal articles, project reports and dissertations. Overall, geo-referenced locations and dam parameters were obtained for a total of 1268 existing dams (out of an estimated total of over 2000 [40]) in SSA (see Additional file 1).

### Estimating reservoir perimeters

Data on reservoir perimeter are necessary to estimate the population at risk of malaria due to a dam. However, these data are not available for most dams, so reservoir perimeter was estimated using a method adapted from Keiser et al. [10]. The method estimated the perimeter based on dam and reservoir characteristics and an assumed rectangular shape. (For more details see Kibret et al. [11]).

### Malaria data

The MAP database [41] assembled all existing malaria surveys to produce a global spatiotemporal malaria dataset at a 1 × 1-km spatial resolution. The data were interpolated within a Bayesian space–time geo-statistical framework to predict the average *P. falciparum* infection rate (PfIR) in the 2020s, 2050s and 2080s using biological models based on estimated average temperature increases in future years. These predictions were calibrated using the 2000–2010 malaria dataset from literature and the WHO [42]. This database was used to estimate the PfIR around dam-associated reservoirs in SSA for the 2020s, 2050s and 2080s in both RCP 2.6 and 8.5.

Non-linear relationships between malaria incidence and climatic predictor variables (i.e., temperature and rainfall) were constructed using a genetic programming method, to predict the spatial distributions of malaria under climate change scenarios. Spatiotemporal scan analysis was performed to identify and quantify the spatiotemporal clustering scales of malaria incidence to empirically estimate the relationship between malaria incidence and climatic factors using the output of the MAP Bayesian statistical model, which combines malaria survey data with climate predictors to provide a gridded malaria incidence for each of the future time intervals. Details of the spatiotemporal malaria incidence prediction procedures are documented in MAP database [41].

The classification described by Gething et al. [43] was used to characterize the epidemiological settings in which the dams were located. These were defined as:

- stable transmission in areas with annual PfIR greater than 0.1 cases per 1000 population;
- unstable transmission in areas with annual PfIR between 0 and 0.1 cases per 1000 population; and
- no malaria in areas having zero annual PfIR.

### Future climate scenarios

Caminade et al. [27] developed malaria maps using a multi-malaria model, which comprised five well-known, process-based, malaria models (i.e., LMM_RO, MIASMA, VECTRI, UMEA, MARA) driven by climate outputs from five global circulation models (GCMs) (i.e., HadGem2-ES, IPSL-CM5A-LR, MIROC-ESM-CHEM, GFDL-ESM2M, NorESM1-M). These all employed bias-corrected temperature and rainfall data from the coupled model intercomparison project phase 5 (CMIP5). A common metric (i.e., length of malaria transmission season) was used to compare all malaria models. Separate maps were produced for each of the four Intergovernmental Panel on Climate Change (IPCC) RCPs: (i.e., RCP 2.6, RCP 4.5, RCP 6.0, RCP 8.5), for three future time intervals (i.e., 2020s, 2050s, 2080s). RCPs are time and space-dependent trajectories of concentrations of greenhouse gases and pollutants resulting from human activities, including changes in land use. RCPs provide a quantitative description of concentrations of the climate change pollutants in the atmosphere over time, as well as their radiative forcing in 2100 (for example, RCP 6 achieves an overall impact of 6 W per sq. m by 2100). Radiative forcing, expressed as Watts per sq. m, is the additional energy taken up by the Earth system due to the enhanced greenhouse effect. The four RCPs illustrate the range of year-2100 radiative forcing values found in the literature, i.e., from 2.6 to 8.5 W per sq. m [44]. Details of the selection process and parametrization are documented in Caminade et al. [27].

Among the four RCPs, two (RCP 2.6 and RCP 8.5) were selected for the current study in order to show how malaria distribution changes between the lowest (i.e., RCP 2.6) and the highest (RCP 8.5). The maps for each RCP and time interval were overlaid separately on the malaria and dam distribution maps to develop future malaria distribution scenarios around dams in the 2020s, 2050s and 2080s.

### Population data

1 × 1-km gridded projected future SSA annual population data were obtained from the International Institute for Applied Systems Analysis (IIASA) for RCP 2.6 and 8.5, for the three future time intervals [45]. In each RCP, the average population in the decade was determined and the data were imported to ArcGIS and overlaid on the dam-malaria-climate distribution maps. The population around dams (i.e., within 5-km radius) was then calculated separately for zones of stable, unstable and no malaria transmission for each time interval and climate scenario. The impact of a dam on malaria was assumed to be negligible beyond 5 km due to mosquitoes’ limited flight range [46]. Also, the population concentration around reservoir relative to non-reservoir communities was assumed constant between 2020s and 2080s.

### Statistical analysis

#### Mapping dams and malaria in climate change scenarios

The locations of dams were overlaid on the malaria-climate scenario maps to develop separate maps for RCP 2.6 and RCP 8.5 in the 2020s, 2050s and 2080s using ArcGIS. For both RCPs, the number of dams in each malaria transmission stability zone (i.e., stable, unstable and no malaria) was counted for each future time interval.

#### Estimating population at risk around dams

Future populations were analysed separately for the two RCPs. It should be noted that future population projections vary between RCPs: the SSA population in the 2080s is estimated to be 1.55 billion and 1.65 billion in RCP 2.6 and 8.5, respectively. The populations at risk of malaria (<5 km) around existing dam-associated reservoirs in areas supporting stable and unstable transmission were estimated for the two RCPs for each of the three time intervals.

#### Estimating future malaria incidence around reservoirs

Using MAP spatiotemporal malaria database, annual PfIRs (hereafter called malaria incidence) for reservoir communities (i.e., located within 5 km of a reservoir shoreline) and non-reservoir communities (i.e., located 5–10 km from a reservoir-shoreline) were estimated for the two RCPs for each of the three time intervals. The annual malaria incidences in reservoir and non-reservoir communities were compared in stable and unstable areas for each RCP in each time interval. Previously documented data of annual malaria incidence for 2010 [11] were used as a baseline against which to measure the degree of future change in malaria incidence. Chi square tests were employed to compare the differences in malaria incidence between malaria stability zones and across time intervals. The same test was carried out to determine whether the difference in malaria incidence between reservoir and non-reservoir communities was significant within each malaria stability zone. Statistical analyses were done using statistical software, SPSS version 22 (SPSS Inc, Chicago, IL, USA). The level of significance was determined at the 95 % confidence interval (*P* = 0.05).

#### Estimating future malaria cases associated with dams

For each time interval, projected annual malaria incidence was multiplied by the population within 5 km and 5–10 km from reservoirs, to estimate the number of malaria cases in reservoir and non-reservoir communities, respectively, in areas of stable and unstable transmission. The number of annual malaria cases attributable to dams was estimated by calculating the difference in the number of annual malaria cases for communities less than 5 km and for communities 5–10 km from the reservoir, allowing for differences in population. The proportion (%) of cases attributable to dams relative to the total malaria cases (<5 km) was also calculated.

#### Identifying the increase in malaria cases attributable only to population increase

To distinguish the increased malaria due to population growth from that associated with climate change, the number of malaria cases due solely to population growth was determined. The 2010 malaria incidence rate was applied to population increases (i.e., the change in population between the 2010 and the average population in future time intervals of the 2020s, 2050s and 2080s for each RCP) to calculate the number of additional malaria cases due to population increases in each of the three periods for each RCP.

#### Identifying the increase in malaria cases attributable only to climate change

For each RCP, the number of cases in 2010 and the number of additional cases associated with population growth were subtracted from the total malaria cases around dams in future time intervals. The results were considered to be the number of cases attributable solely to climate change. The difference in socio-economic and other environmental factors was assumed to be minimal between reservoir and non-reservoir communities. Possible impacts of further malaria control interventions and other non-climate factors (e.g., socio-economic development) in the future were not captured.

## Results

### Spatial distribution of dams in future years as a consequence of changing climate

**Fig. 1:**
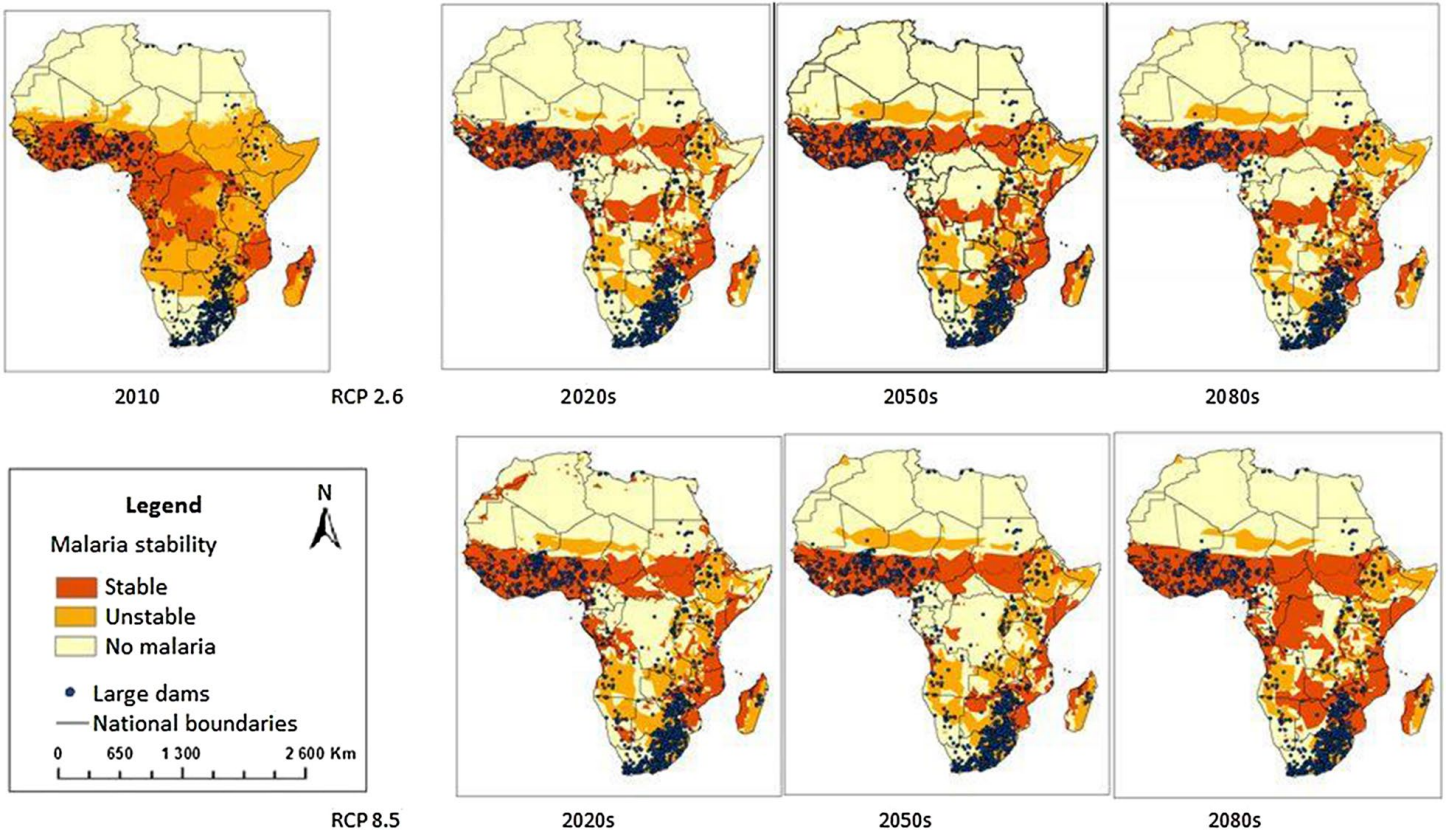
Distribution of large dams in sub-Saharan Africa in relation to malaria stability in 2010, 2020s, 2050s and 2080s using two future climate scenarios (RCPs) (Adopted from Caminade et al. [27])

Depending on the RCP, the total area suitable for stable malaria transmission is projected to increase from 7 million sq. km in 2010 to 9–11 million sq. km in the 2080s across SSA (Fig. 1; Table 1). Concurrently, the area supporting unstable transmission is predicted to shrink from 14 million sq. km in 2010 to 11–13 million sq. km in the 2080s and the non-malarious area is expected to decline from two million sq. km to 1.1–1.5 million sq. km over the same period. Between 2010 and the 2080s, the number of existing dams located in areas suitable for stable transmission is projected to increase from 307 currently, to 386 (i.e., by 20 %) and to 427 (i.e., by 30 %) in RCP 2.6 and RCP 8.5, respectively. Similarly, by the 2080s, the number of dams located in areas with unstable transmission is projected to increase from 416 to 640 (i.e., by 35 %) in both RCPs. In contrast, the number of dams located in areas with no malaria is predicted to reduce by 50–65 % in the 2080s depending on the RCP, under the assumption that control measures have no further impact.

**Table 1:** Summary of number of dams and projected average number of people living around dams in the 2020s, 2050s and 2080s in stable, unstable and no malaria zones across sub-Saharan Africa, using two future climate scenarios (RCP 2.6 and RCP 8.5) (Data for 2010 is included for comparison: source: Kibret et al. [11])

	2010				2020s				2050s				2080s			
	Area (sq. km)	Population in the region	No. dams (%)	No. people within 5 km from reservoirs	Area (sq. km)	Population in the region	No. dams	No. people within 5 km from reservoirs	Area (sq. km)	Population in the region	No. dams (%)	No. people within 5 km from reservoirs	Area (sq. km)	Population in the region	No. dams (%)	No. people within 5 km from reservoirs
RCP 2.6																
Stable	7,086,268	298,682,666	307 (24)	6,406,226	7,408,321	361,264,297	354 (28)	7,198,341	8,264,718	489,811,003	377 (30)	8,872,392	9,113,482	546,622,885	386 (30)	10,097,454
Unstable	14,459,409	418,451,433	416 (33)	8,193,239	14,378,791	462,113,462	433 (34)	9,202,466	13,666,490	590,443,015	645 (51)	12,105,161	12,972,969	682,788,932	628 (50)	14,713,709
No malaria	2,042,223	273,355,662	545 (43)	5,605,474	1,800,788	322,401,006	481 (38)	4,891,743	1,656,692	281,714,037	246 (19)	4,048,543	1,501,449	319,587,199	254 (20)	3,310,021
Total	23,587,900	990,489,781	1268 (100)	20,204,939	23,587,900	1,145,778,765	1268 (100	21,292,550	23,587,900	1,361,968,055	1268 (100	25,026,096	23,587,900	1,548,999,016	1268 (100)	28,121,184
RCP 8.5																
Stable	7,086,268	298,682,666	307 (24)	6,406,226	7,871,237	403,671,332	381 (30)	8,671,114	8,671,114	498,594,276	346 (27)	9,408,302	10,945,711	572,083,572	427 (34)	10,983,666
Unstable	14,459,409	418,451,433	416 (33)	8,193,239	13,950,337	501,242,107	609 (48)	10,309,672	10,309,672	621,144,735	692 (55)	13,611,448	11,511,801	697,130,875	645 (51)	15,231,541
No malaria	2,042,223	273,355,662	545 (43)	5,605,474	1,766,326	388,923,354	268 (21)	3,833,107	3,833,107	349,366,669	230 (18)	3,115,432	1,130,388	381,136,413	196 (15)	3,109,886
Total	23,587,900	990,489,781	1268 (100)	20,204,939	23,587,900	1,293,836,793	1268 (100)	22,813,893	23,587,900	1,469,105,680	1268 (100)	26,135,182	23,587,900	1,650,350,860	1268 (100)	29,325,093

### Estimated future population at risk of malaria around dams in SSA

The number of people living in close vicinity to the 1268 dam associated reservoirs (<5 km) is projected to increase steadily over the period to 2080s, with a considerable increase in both stable and unstable transmission areas (Table 1). The total population living within 5 km of the reservoirs is estimated to increase from 20 million in 2010 to 21–23 million in the 2020s, 25–26 million in the 2050s and 28–29 million in the 2080s, depending on the RCP. The population at risk of malaria is substantially greater in RCP 8.5 scenario than RCP 2.6. In areas of stable transmission, the population within 5 km of reservoirs is projected to increase from 6.4 million in 2010 to 7.2–8.7 million in the 2020s, 8.9–9.4 million in the 2050s and 10.1–11.0 million in the 2080s depending on RCP. Similarly, the population within 5 km in areas of unstable transmission is predicted to increase from 8.2 million in 2010 to 9.2–10.3 million in the 2020s, 12.1–13.6 million in the 2050s and 14.7–15.2 million in the 2080s depending on the RCP. Overall, the population at risk of malaria is estimated to be consistently higher in areas of unstable than stable transmission in both RCPs.

### Estimated malaria incidence around dams in future climate in SSA

**Fig. 2:**
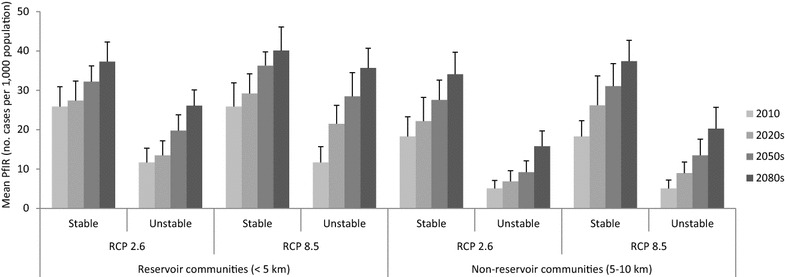
Estimated average annual malaria incidence in the 2020s, 2050s and 2080s in communities living close to (<5 km) and further away (5–10 km) from dam-associated reservoirs in sub-Saharan Africa, using two future climate scenarios (RCP 2.6 and RCP 8.5). Data for 2010 is included for comparison: source Kibret et al. [11]

Annual malaria incidence (cases per 1000 population per year) around existing dams (i.e., <5 km) in SSA is projected to increase in the future in areas of both stable and unstable transmission (Fig. 2). The annual malaria incidence in reservoir communities situated in areas of stable transmission is expected to increase by an average of 30 % between 2010 and the 2080s in both RCPs, but this increase was not statistically significant (χ^2^ = 6.212; df = 2; *P* = 0.061). However, in areas of unstable transmission, the annual malaria incidence is likely to increase twofold to threefold in the 2080s when compared to 2010 and this difference was statistically significant (χ^2^ = 13.061; df = 2; *P* < 0.001) in both RCPs.

### Annual malaria cases associated with dams in the future

In future years, depending on the RCP, the proportion of malaria cases attributable to dams relative to all cases around dams will be 19–61 % and 50–72 % in areas of stable and unstable transmission, respectively (Table 2). For both RCPs, the number of annual malaria cases around reservoirs in areas of stable transmission is projected to triple to 600,000–700,000 in the 2080s compared to 2010. The number of cases associated with dams in areas with unstable transmission is estimated to increase from 919,000 in 2010 to 1.7–2.6 million in the 2080s depending on the RCP. Thus, in the absence of improved mitigating measures, dams in SSA are expected to add 1.2–1.6 million malaria cases annually in the 2020s and 2.4–3.0 million cases annually in the 2080s. The malaria impact of dams is projected to be 1.8–2.3 (χ^2^ = 18.903; df = 2; *P* < 0.001) and 2.1–4.6 (χ^2^ = 33.615; df = 2; *P* < 0.001) times higher in unstable than stable areas across the future years in RCP 2.6 and RCP 8.5, respectively.

**Table 2:** Estimated annual malaria cases around large dams in sub-Saharan Africa in the 2020s, 2050s and 2080s using two future climate scenarios (RCP 2.6 and RCP 8.5) (Data for 2010 is presented for comparison: source: Kibret et al. [11])

Climate scenario/malaria stability	2010		2020s			2050s			2080s		
	Number malaria cases <5 km	Number malaria cases attributable to presence of dams	Number malaria cases <5 km	Number malaria cases (assuming similar case rate as >5 km)	Number malaria cases attributable to presence of dams	Number malaria cases <5 km	Number malaria cases (assuming similar case rate as >5 km)	Number malaria cases attributable to presence of dams	Number malaria cases <5 km	Number malaria cases (assuming similar case rate as >5 km)	Number malaria cases attributable to presence of dams
RCP 2.6											
Stable	1,337,979	203,718	2,221,953	1,794,093	427,860	3,008,238	2,369,683	638,555	3,763,321	3,006,427	718,070
Unstable	1,337,956	919,281	1,204,302	416,290	788,012	2,402,874	937,677	1,465,197	3,052,593	1,660,640	1,656,318
Total	2,675,935	1,122,999	3,426,255	2,210,383	1,215,872	5,411,112	2,307,360	2,103,752	6,815,914	5,067,067	2,374,388
RCP 8.5											
Stable	1,337,979	203,718	2,507,720	1,905,353	602,367	3,162,273	2,312,261	850,013	4,007,444	3,406,427	601,017
Unstable	1,337,956	919,281	1,624,363	535,513	1,088,850	4,051,684	1,919,967	2,131,717	4,976,346	2,606,657	2,369,689
Total	2,675,935	1,122,999	4,132,083	2,520,866	1,611,217	7,213,957	4,232,228	2,981,730	8,983,790	6,013,084	2,970,706

### Impacts of population growth

The number of additional malaria cases around dams due to population growth is expected to increase in the future in both RCPs (Table 3). In stable areas, the average annual number of malaria cases around dams due to population growth is estimated to be 205,000–587,000 in 2020s, 639,000–777,000 in 2050s and 956,000–1.2 million in 2080s. In unstable areas, additional malaria cases due to population rise is estimated to increase: from 118,000–148,000 in the 2020s to 458,000–634,000 in the 2050s and 763,000–823,000 in the 2080s depending on RCP.

**Table 3:** Increases in malaria cases around SSA dams due to population growth and climate change in 2020s, 2050s and 2080s in two RCPs (RCP 2.6 and RCP 8.5)

	2020s			2050s			2080s		
	Additional malaria cases due to population increase (%)^a^	Additional malaria cases due to climate change (%)	Total additional malaria cases due to population increase and climate change	Additional malaria cases due to population increase (%)	Additional malaria cases due to climate change (%)	Total additional malaria cases due to population increase and climate change	Additional malaria cases due to population increase (%)	Additional malaria cases due to climate change (%)	Total additional malaria cases due to population increase and climate change
RCP 2.6									
Stable	205,158 (42)	281,234 (58)	486,392	638,737 (57)	491,522 (43)	1,130,259	956,028 (40)	1,429,314 (60)	2,385,342
Unstable	118,080 (46)	136,618 (54)	254,698	457,695 (43)	607,223 (57)	1,064,918	762,895 (44)	951,742 (56)	1,714,637
Total	323,238 (41)	457,851 (59)	781,089	1,096,432 (50)	1,098,745 (50)	2,195,177	1,718,923 (42)	2,381,056 (58)	4,099,979
RCP 8.5									
Stable	586,606 (61)	373,335 (39)	959,941	777,538 (43)	1,046,756 (57)	1,824,294	1,185,557 (44)	1,483,908 (56)	2,669,465
Unstable	147,623 (43)	198,784 (57)	346,407	633,930 (28)	1,634,556 (72)	2,268,486	823,481 (27)	2,200,442 (73)	3,023,923
Total	834,229 (59)	572,119 (41)	1,406,348	1,411,468 (34)	2,681,312 (66)	4,092,780	2,009,038 (35)	3,684,350 (65)	5,693,388

^a^% is calculated from total additional cases due to population increase and climate change

### Increases in malaria cases around dams attributable to climate change

The annual number of malaria cases around 1268 existing dams that is attributable to climate change is projected to increase in the future in both RCPs (Table 3). In areas of stable transmission, the annual number of malaria cases around dams due to climate change is estimated to be 281,000–373,000 in the 2020s, 491,000–1.0 million in the 2050s and 1.4–1.5 million in the 2080s depending on the RCP. By comparison, the number of malaria cases due to climate change in areas of unstable transmission is estimated to be 137,000–199,000 in the 2020s, 607,000–1.6 million in the 2050s and 952,000–2.2 million in the 2080s. The number of cases is generally expected to be higher in RCP 8.5 than RCP 2.6 scenario. The relative impact of climate change on malaria transmission is projected to be greater than the impact of population growth in all time intervals (Table 3). Note that total number of malaria cases in Tables 2 and 3 are not equivalent because while Table 2 presents the number of cases attributable to the presence of a dam, the figures in Table 3 show the number of cases due to climate change and population growth.

## Discussion

### Malaria transmission around dams in SSA to more than double by 2080s

The present findings have reinforced and amplified pre-existing concerns related to current and future malaria transmission around dams in SSA. Assuming no change in socio-economic and disease intervention efforts, the dam-associated malaria burden in the vicinity of 1268 dams in SSA is projected to increase from 1.1 million in 2010 to 2.4–3.0 million in the 2080s. The population at risk of malaria around these dams is set to increase from 15 million in 2010 [11] to 24–25 million in the 2080s. This study is the first of its kind to project the expected malaria burden associated with dams under future climatic conditions. The estimates are conservative because no allowance was made for the approximately 800 dams that have been built but for which geo-referenced locations were unavailable. Furthermore, future dam construction, also unaccounted for here, will exacerbate the trends.

### Climate change exacerbates malaria around dams

The present study confirmed that the role of climate change on malaria around dams will be substantial. Considerable number of malaria cases was associated with climate change and population growth and these are generally higher in the unstable than stable malarious areas. Zhou et al. [47] found a high spatial variation in the sensitivity of malaria case number to climate fluctuation in the highlands of East Africa, where major dams are located. Thus, climate change could further exacerbate the impact of dams on malaria.

### Malaria impact of climate change around dams is consistent with malaria impacts associated with future regional climate scenarios

Malaria is expected to increase in the future climate in the highland regions throughout Africa, including South Africa and Zimbabwe where more than half of SSA’s current large dams are located. Ebi et al. [48], for example, projected an increase in the malaria suitability of Zimbabwean highlands. Likewise, Caminade et al. [27] indicated an increase in the length of the malaria transmission season over the high altitude regions of Ethiopia, Kenya, Madagascar, South Africa, and Angola. Peterson [49] and Ermert et al. [50] predicted an increase in the suitability of the East African highlands for malaria by 2050. Siraj et al. [51] predicted that a 1 °C increase in daily mean temperature will result in three million additional malaria cases in the unstable transmission highlands of Africa. Spatial analyses by Tonnang et al. [52] indicated that malarious areas will expand southwards and eastwards of Africa in future climate scenarios. For most of these regions, climate becomes suitable in the future as a result of predicted temperature rises.

### Relative impact of dams *vs* climate change: interpreting attribution

This study determined the number of malaria cases attributable to dams in the future as well as the number of cases in the vicinity of dams that are attributable to climate change. Comparison of such numbers reveals two things. First, while the impacts of dams are greater in the 2020s, the relative impact of climate change around dams increases over time such that the malaria impacts associated with climate change overtake those due to dams by the 2080s. Second, despite this trend toward greater climate change impacts, dams generally remain a greater risk factor than climate change in areas of unstable malaria transmission.

### Greater impacts in a smaller area: explaining a paradox

The present study found that the rate of malaria transmission around dams in unstable areas increases despite the fact that the aggregate size of unstable areas decreases. This seemingly paradoxical future is explained by the possibility that relatively dam-dense areas, with no malaria transmission at present, could transition to regions of unstable transmission due to climate change. Particular areas of concern for emerging malaria around dams are South Africa, Zimbabwe and highland areas in Ethiopia and Kenya. The influence of dams on malaria could be of concern in such areas given that populations will often lack any immunity to the disease. Conversely, the magnitude of such impact may not be easily felt in countries like South Africa where strong malaria control programmes exist.

### Malaria around dams in the context of broader efforts towards malaria reduction

Despite the ongoing efforts to develop models to predict the impacts of future climate, there remains notable uncertainty in their robustness. The malaria models adopted in the present study [27] did not incorporate socio-economic, land use, disease interventions (e.g., bed net usage), and other environmental factors (e.g., topography), all of which influence the distribution and intensity of malaria in SSA. Gething et al. [53], for example, shows that despite a climate that warmed over the twentieth century, malaria has declined globally due to economic growth and implementation of control programmes. Although the degree of future support of malaria control in SSA is uncertain [31], it must be recognized that the malaria projections will not necessarily translate into actual changes in malaria risk. Ultimately, such evidence of past progress should serve to encourage renewed emphasis on control in order to help ensure the potential increased risk associated with climate change and dams is mitigated, and transmission of the disease is indeed *rolled back*. Targeted intensification of malaria interventions may certainly curb a considerable amount of the potential malaria increase [53].

### The evolving nature of malaria transmission stability around dams: implications for control efforts

Given the increasing evidence of greater malaria impacts of dams in unstable areas, and the fluid nature of zones of malaria transmission stability, valid questions may be posed about how to best match the degree of control to the degree of threat. It would seem that in areas predicted to transition from unstable to stable transmission, dams will gradually shift to wielding relatively less of an impact on surrounding communities. As such, control efforts in such areas may progressively diversify away from heavy focus on the dam to a broader set of risk factors. Conversely, in areas transitioning from no malaria to unstable malaria, the impacts of dams could be disastrous as communities and health systems are less likely to be equipped to cope with the disease. In these areas, it will be critical to progressively roll out control programs as the disease transmission spreads. To achieve the significant decreases proposed by the Roll Back Malaria Partnership [32], foresight must be applied so that limited disease intervention resources are optimally targeted.

### Climate-proofing malaria control around dams

This study determined that the threat of adverse impacts from dams in SSA will grow in future years. As areas of unstable transmission expand in eastern and southern Africa, at least two modifications to malaria intervention efforts around dams may be needed to sustain the current advances in reducing transmission of the disease. First, health impact assessments (HIAs) that are routinely conducted for dams should consider malaria impacts today and tomorrow. In particular, risks associated with future malaria transmission should be clearly flagged for dams constructed in ‘fringe areas’, presently outside of the zone of transmission but in which malaria is predicted to expand. Second, the amplification of disease transmission, and uncertainty associated with the altered pathways of transmission, may be best mitigated with a diversified approach to control that draws on both conventional and unconventional measures. Current malaria interventions mainly rely on bed nets and indoor residual spraying. Alternative control measures, such as reservoir management that suppress shoreline-associated mosquito breeding habitats, have previously been used in the western world and proved to be effective and economically viable [54, 55]. However, reservoir management has never been used as a malaria control tool in Africa despite growing evidence of its potential [56–58]. Measures such as this, focused on mosquito larval management, could supplement existing malaria interventions.

### Caveats

Three methodological limitations should be pointed out. First, the study did not include about one-third of currently existing dams due to their lack of geo-referenced data. Second, the study did not consider planned dams. Failing to incorporate more than one-third of existing dams, and all future dams, means that: (i) modelled predictions of future malaria transmission are likely to underestimate actual transmission, and, (ii) the magnitude of impact of dams across regions and malaria transmission zones may be somewhat distorted. Third, the potential impact of control efforts was not included as a covariate. While incorporating potential impacts of control may have helped to achieve a more accurate depiction of future conditions, predicting the degree of malaria reduction associated with interventions around dams that is achieved and sustained is not straightforward. Evidence on intervention impacts around dams is indeed scant, and evidence on sustainability of such impacts is non-existent. As such, quantification of the disease reduction resulting from interventions around dams could not be confidently undertaken.

### Future research

The present study only examined the potential malaria risk associated with future climate scenarios around large dams in SSA using population and climate data. More localized studies should explore malaria risk associated with climate change in the vicinity of dams using robust data on socio-economic, disease intervention, vector distribution, and other environmental variables. Future investigations need also to assess how temperature and rainfall changes will affect run-off, future dam development, and hence future dam operations. Understanding this broader picture will in turn provide a more holistic understanding of dam impacts on malaria transmission.

More broadly, future work will do well to identify and address structural bottlenecks for incorporating malaria control into water resources planning and management. Notably, the present findings add to a growing body of evidence (cited in the “Background” of this paper) on malaria transmission around large dams in Africa. Nevertheless, the degree to which relevant policies and management approaches have been modified in light of this evidence is debatable. While the need for intersectoral collaboration has indeed been widely highlighted as a suggested response option [59], the lack of action in this regard calls for greater scrutiny into institutional constraints for operationalization.

### More dams in the future

Moving forward, dam building in Africa will continue and may even accelerate. In 2012, the continent’s heads of state and government laid out an ambitious, long-term plan for closing Africa’s infrastructure gap [60]. In the water and power sector, the Programme for Infrastructure Development in Africa (PIDA) calls for an expansion of hydroelectric power generating capacity by more than 54,000 MW and of water storage capacity by 20,000 cubic km. In response to this, Ethiopia has built several dams in recent years that have helped satisfy its growing energy demand, and the country is currently building an additional eight large dams, including the huge Grand Renaissance Dam [61]. In central Africa, the Democratic Republic of Congo has initiated the construction of several dams along the Congo River at Inga Falls [62]. In West Africa, several dams have been planned or are under construction, including the Fomi Dam in Guinea, Toussa Dam in Mali, and Kandadji Dam in Niger [63]. In southern Africa, the height of the Kamuzu barrage is being augmented in Malawi, and feasibility studies have identified lucrative hydropower potential on the Ruo, which straddles the Malawi-Mozambique border [64]. These extensive dam constructions should therefore be carefully analysed so that proper malaria mitigation measures can be implemented while designing and operating them.

## Conclusions

The findings of this study highlight the potential for climate change to significantly add to the malaria burden in the vicinity of large dams in SSA. There is a need for upstream planning and diversification of control efforts in order to cope with the climate change impacts of malaria around dams. Future dam constructions should consider optimizing dam management to incorporate malaria control measures during design and operation. Although dams remain extremely important for socio-economic development, the potential amplification of their adverse health impacts calls for smarter approaches built on a foundation of collaboration between water managers and health authorities.

## Additional file

10.1186/s12936-016-1498-9 List of existing African dams used in the study. The data presents the location of the 1268 dams used in the present study.

## Abbreviations

CMIPS: coupled model intercomparison project phase 5
df: degree of freedom
FAO: Food and Agriculture Organization
GCM: global circulation model
GRanD: global reservoirs and dams
HIA: health impact assessment
IIASA: International Institute for Applied Systems and Analysis
IPCC: Intergovernmental Panel on Climate Change
MAP: malaria atlas project
MW: megawatts
PfIR: *Plasmodium falciparum* infection rate
PIDA: programme for infrastructure development in Africa
RCP: representative concentration pathways
SSA: sub-Saharan Africa
WHO: World Health Organization
